# The role of daily adjustment disorder, depression and anxiety symptoms for the physical activity of cardiac patients

**DOI:** 10.1017/S0033291722003154

**Published:** 2023-10

**Authors:** Tania Bermudez, Andreas Maercker, Walter Bierbauer, Artur Bernardo, Ruth Fleisch-Silvestri, Matthias Hermann, Jean-Paul Schmid, Urte Scholz

**Affiliations:** 1 Applied Social and Health Psychology Unit, University of Zurich, Zurich, Switzerland; 2 Department Health Science, Institute of Sport Science, University of Bern, Bern, Switzerland; 3 University Research Priority Program ‘Dynamics of Healthy Aging’, University of Zurich, Zurich, Switzerland; 4 Psychopathology and Clinical Intervention Unit, University of Zurich, Zurich, Switzerland; 5 Clinic Gais AG, Gais, Switzerland; 6 Clinic Schloss Mammern, Mammern, Switzerland; 7 Department of Cardiology, University Heart Center Zurich, University Hospital Zurich, Zurich, Switzerland

**Keywords:** Adjustment disorder, cardiac rehabilitation, depression and anxiety, physical activity, sedentary behaviour

## Abstract

**Background:**

Physical activity (PA) is crucial in the treatment of cardiac disease. There is a high prevalence of stress-response and affective disorders among cardiac patients, which might be negatively associated with their PA. This study aimed at investigating daily differential associations of International Classification of Diseases (ICD)-11 adjustment disorder, depression and anxiety symptoms with PA and sedentary behaviour (SB) during and right after inpatient cardiac rehabilitation.

**Methods:**

The sample included *N* = 129 inpatients in cardiac rehabilitation, *M*_age_ = 62.2, s.d._age_ = 11.3, 84.5% male, *n* = 2845 days. Adjustment disorder, depression and anxiety symptoms were measured daily during the last 7 days of rehabilitation and for 3 weeks after discharge. Moderate-to-vigorous PA (MVPA), light PA (LPA) and SB were measured with an accelerometer. Bayesian lagged multilevel regressions including all three symptoms to obtain their unique effects were conducted.

**Results:**

On days with higher adjustment disorder symptoms than usual, patients engaged in less MVPA, and more SB. Patients with overall higher depression symptoms engaged in less MVPA, less LPA and more SB. On days with higher depression symptoms than usual, there was less MVPA and LPA, and more SB. Patients with higher anxiety symptoms engaged in more LPA and less SB.

**Conclusions:**

Results highlight the necessity to screen for and treat adjustment disorder and depression symptoms during cardiac rehabilitation.

## Theoretical background

Cardiac disease is the leading cause of death and disability worldwide (Khan et al., 2020). It is mainly caused by lifestyle risk factors, such as physical *inactivity*, which is the fourth leading risk factor for mortality worldwide (World Health Organization – WHO, 2017). Moreover, regular physical activity (PA) independently decreases the risk of cardiac events (Varghese et al., 2016). Therefore, regular PA is paramount in the prevention and treatment of cardiac disease. Consequently, one of the main aims of cardiac rehabilitation programmes is to help patients achieve the recommended amount of PA in everyday life (Bierbauer et al., 2020). Exercise-based cardiac rehabilitation programmes are successful in improving exercise capacity, and reducing the risk of cardiovascular mortality and hospital admissions (Anderson et al., 2016; Bierbauer et al., 2020). Nevertheless, there are mixed results regarding the short- and long-term adherence of patients to PA recommendations (Ter Hoeve et al., 2015). Therefore, it is crucial to identify relevant factors for the PA of patients as inpatient cardiac rehabilitation offers an ideal opportunity for intervention (Pogosova et al., 2015). Stress-response and affective disorders are highly prevalent among cardiac patients and might represent detrimental factors for PA (Edmondson & von Känel, 2017; Pengpid & Peltzer, 2019). Nevertheless, adjustment disorder and its association with the PA of patients have been neglected in the literature, even though symptoms of adjustment disorder emerge as a reaction to an identifiable stressor, such as cardiac disease (Maercker & Lorenz, 2018).

### Adjustment disorder symptoms

Cardiac patients can be expected to be at particular risk of adjustment disorder, given that cardiac disease constitutes an important life stressor (De Ridder, Geenen, Kuijer, & van Middendorp, 2008). Nevertheless, there is little and unreliable research pertaining adjustment disorder as its diagnostic definition was rather unspecific in the 10th revision of the International Classification of Diseases (ICD-10; WHO, 1992; Maercker & Lorenz, 2018). Consequently, a more specific diagnostic definition was developed for the 11th revision (ICD-11; WHO, 2018; Kazlauskas, Zelviene, Lorenz, Quero, & Maercker, 2017; Maercker & Lorenz, 2018). Symptoms ought to emerge within a month after an identifiable stressor occurred. Chronic diseases can represent such a stressor (De Ridder et al., 2008). ICD-11 adjustment disorder has two core symptoms: preoccupations and failure to adapt (Levin et al., 2022; Maercker & Lorenz, 2018). In the case of cardiac disease, *preoccupations* can comprise repeated, afflicting, and involuntary thoughts about the disease or its consequences (e.g. own ageing or mortality); as well as rumination or stress as a reaction to recollections of circumstances surrounding the disease (e.g. a cardiac event). *Failure to adapt* can manifest as concentration and sleep difficulties, as lack of self-confidence in familiar activities and/or as loss of interest in work, social life, the care for other people and/or leisure activities. Given that these new diagnostic criteria are quite different from ICD-10 (Maercker et al., 2013), there is no research on the relationship between adjustment disorder symptoms and PA to date. Nevertheless, a relationship can be expected, because other stress-response syndromes have adverse effects on PA (Edmondson & von Känel, 2017), and show negative associations with determinants of health behaviour (Luszczynska, Benight, & Cieslak, 2009). Moreover, ICD-10 adjustment disorder is associated to low perceived behavioural control (Bley, Einsle, Maercker, Weidner, & Joraschky, 2008), a prominent predictor of PA (Steinmetz, Knappstein, Ajzen, Schmidt, & Kabst, 2016).

### Depression and anxiety symptoms

Depression and anxiety symptoms are not only possible accessory symptoms of ICD-11 adjustment disorder (Maercker & Lorenz, 2018), but in general highly prevalent among cardiac patients (Rao et al., 2020). For example, a study found a prevalence of 18% and 28% of moderate depression and anxiety accordingly among patients entering cardiac rehabilitation in Australia (Rao et al., 2020). Indeed, depression and anxiety seem to share a common epidemiology with cardiovascular disease (Wong et al., 2019). Biological and behavioural mechanisms have been proposed to explain the relationship between affective psychopathology and cardiovascular outcomes (Machado et al., 2018), including physical inactivity (Pengpid & Peltzer, 2019). Furthermore, depression and anxiety have been linked to reduced self-management behaviours in cardiac patients (Fredericks, Lapum, & Lo, 2012). Empirical research has consistently found a negative relationship between depression and PA in general (Schuch et al., 2017), and among cardiac patients in particular (Prugger et al., 2016; Whooley et al., 2008). Baseline depression predicts the later development of a sedentary lifestyle (Roshanaei-Moghaddam, Katon, & Russo, 2009).

The literature pertaining the association between anxiety symptoms and PA is less consistent. Some studies found a negative association of anxiety with PA (Azevedo Da Silva et al., 2012; Strine, Chapman, Kobau, & Balluz, 2005). However, research has predominantly focused on the other direction of this association with a meta-analysis finding a small negative effect size of PA on anxiety (Rebar et al., 2015). There is also evidence of bidirectionality (Azevedo Da Silva et al., 2012). Thus, based on these findings a negative association between anxiety symptoms and PA could be expected. Nevertheless, studies also found a positive effect of general anxiety disorder on cardiac outcomes (Parker, Hyett, Hadzi-Pavlovic, Brotchie, & Walsh, 2011), as well as positive associations of anxiety with improvements in exercise capacity and in physical quality of life during inpatient cardiac rehabilitation, which are the basis for regular PA in everyday life (Bermudez, Bierbauer, Scholz, & Hermann, 2021a).Therefore, there is also evidence supporting a positive association of anxiety symptoms with PA.

### The present study

Studies investigating the associations described above have exclusively focused on analysing differences between persons (interindividual level). However, it is crucial to also investigate the associations within individuals over time (intraindividual level), because inter- and intraindividual findings do not necessarily coincide (Bolger & Laurenceau, 2013), which prohibits drawing conclusions from interpersonal results to individuals. In line with this so-called ecological fallacy (Sedgwick, 2015), differences have already been found between both levels of analyses in behavioural determinants for PA (e.g. Bermudez et al., 2021b). Thus, examining the relationship of affective and stress-related symptoms with PA should include both the inter- and the intraindividual level. To achieve this, the study used ambulatory assessments of PA (through accelerometry) and daily self-reported adjustment disorder, depression and anxiety symptoms.

Ambulatory and objective assessments of PA and sedentary behaviour (SB) have several strengths. For example, ecological validity is high thanks to assessing data in real-time and in a natural setting (see state of the science review by Reichert et al., 2020). Thus, with daily device-based ambulatory assessments we can capture PA and SB in the everyday life of patients with minimal disruption. By doing so, we also avoid important limitations of self-reports, such as retrospective and recall bias (Reichert et al., 2020). Furthermore, whereas accelerometer ambulatory assessments of PA and SB are associated with physical parameters (e.g. BMI), this is often not the case for PA/SB measured with traditional questionnaires (Ferrari et al., 2020). Also well-established in the field of psychopathology, a recent review highlights the ecological validity, reducing recall bias and intraindividual analyses as current strengths of using ambulatory assessments by, for example, applying daily diaries (Mestdagh & Dejonckheere, 2021). Furthermore, given the affective nature of adjustment disorder, depression and anxiety symptoms and the high intraindividual variability of affect (e.g. Röcke, Li, & Smith, 2009), ambulatory assessments are particularly suited for the present study's aims.

Another big gap in the literature pertains the time dynamics of health behaviour processes (Scholz, 2019). Time dynamics are a highly complex topic as many different time resolutions can be investigated. Nevertheless, it is crucial for research to start deepening our understanding on time dynamics as this could help informing more effective interventions (e.g. just-in-time-adaptive interventions; Scholz, 2019). To start moving in this direction, the intraindividual associations will be investigated both in same-day (today's predictor on today's outcome) and in lagged (yesterday's predictors on today's outcome) analyses. For these purposes, we derived the following hypotheses (registered prior to data analysis: https://osf.io/mkhne):

H1.1. Patients with higher *adjustment disorder* symptoms will be less physically active than patients with lower symptoms (interindividual level).

H1.2. On days with higher *adjustment disorder* symptoms than usual, PA will be shorter (same-day intraindividual level).

H1.3. On days with higher *adjustment disorder* symptoms than usual, tomorrow's PA will be shorter (lagged intraindividual level).

H2.1. Patients with higher *depression* symptoms will be less physically active than patients with lower symptoms (interindividual level).

H2.2. On days with higher *depression* symptoms than usual, PA will be shorter (same-day intraindividual level).

H2.3. On days with higher *depression* symptoms than usual, tomorrow's PA will be shorter (lagged intraindividual level).

H3a.1. Patients with higher *anxiety* symptoms will be *less* physically active than patients with lower symptoms (interindividual level).

H3b.1. Patients with higher *anxiety* symptoms will be *more* physically active than patients with lower symptoms (interindividual level).

H3a.2. On days with higher *anxiety* symptoms than usual, PA will be *shorter* (same-day intraindividual level).

H3b.2. On days with higher *anxiety* symptoms than usual, PA will be *longer* (same-day intraindividual level).

H3a.3. On days with higher *anxiety* symptoms than usual, tomorrow's PA will be *shorter* (lagged intraindividual level).

H3b.3. On days with higher *anxiety* symptoms than usual, tomorrow's PA will be *longer* (lagged intraindividual level).

In general, cardiac patients are recommended to engage in at least 150 min of moderate-to-vigorous PA (MVPA) per week (WHO, 2010). Indeed, the cardiovascular health benefits of regular MVPA are well documented (Pinckard, Baskin, & Stanford, 2019). However, light PA (LPA) is rarely considered and reduces cardiovascular risk even after adjusting for MVPA (Amagasa et al., 2018). Therefore, we investigate the hypotheses for both MVPA and LPA. Furthermore, SB represents a cardiovascular risk independent of PA (Wilmot et al., 2012). A study even found the health benefits of MVPA only, when SB was not excessive (Halloway, Wilbur, Schoeny, Semanik, & Marquez, 2016). Therefore, we also investigate SB as an outcome of the different symptoms (not preregistered).

A final aim of this study involves the effect the context might have on the hypothesized relationships. Whereas during rehabilitation patients receive a structured exercise programme and guidance, they most likely must rely on their self-regulation after discharge to adhere to PA recommendations. Thus, a last aim of the study is to explore whether each hypothesized association differs between both contexts.

## Methods

The present study is part of the larger CAMP-project (Cardiac rehabilitation, Adjustment disorder, Medication adherence, and Physical activity), which had a multi-centric intensive-longitudinal observational design. All information about the study design and procedure is described on https://osf.io/4eqdj. The Cantonal Ethics Committee of Zurich (KEK; REQ-2017-005-08) cleared the project. This study focuses on the diary phase, which started around 7 days before discharge from inpatient cardiac rehabilitation, and continued through the remaining rehabilitation and for 3 more weeks at home. During this time, patients were instructed to fill out short daily questionnaires before bed and to wear an accelerometer on their hip during waking hours. Thus, there could be up to 29 days of data collected per patient (7 days at the clinic, 1 day of discharge and 21 days at home).

In Switzerland, inpatient cardiac rehabilitation includes an exercise programme adapted to the patient's capacity following the quality requirements of the Swiss Working Group for Cardiovascular Prevention, Rehabilitation and Sports Cardiology (https://www.scprs.ch/). The multidisciplinary programme has a recommended duration of 3–4 weeks, including a minimum of 21 therapy sessions each week (endurance training, coordination/balance, outdoor activities, strength training and, in some patients, inspiratory muscle training and relaxation). Patients also receive psychoeducation and counselling regarding cardiovascular risk factors and psychosocial well-being.

### Sample

The initial sample comprised *N* = 156 cardiac patients participating in one of four Swiss inpatient cardiac rehabilitation centres (participant flow of the CAMP-project: https://osf.io/fyv6z). A total of *n* = 27 patients had insufficient data for the preregistered analyses (see registration: https://osf.io/mkhne) and were excluded. Note that only patients with at least one day of data during rehabilitation and one at home were included. The included sample did not significantly differ from the excluded patients in terms of age, *t*(154) = 0.49, *p* = 0.63; gender, χ^2^(1) = 0.01, *p* = 0.93; adjustment disorder symptoms, *t*(152) = −1.05, *p* = 0.29; depression symptoms, *t*(152) = −0.35, *p* = 0.73; anxiety symptoms, *t*(152) = 0.08, *p* = 0.93; MVPA, *t*(145) = 1.64, *p* = 0.10; or LPA, *t*(145) = −0.72, *p* = 0.47. However, the included sample engaged in more SB, *t*(145) = −2.03, *p* = 0.04, *d* = 0.51. The final sample comprised *n* = 129 cardiac patients. Table 1 shows the sample characteristics.

**Table 1.** Sample characteristics

	*n*	%	*M*	s.d.	Range
Days of data					
Complete days of data	2845	78.8	–	–	–
Days of data per patient	–	–	22.05	5.82	2–29
Days during rehabilitation	–	–	4.89	1.79	1–7
Days at home	–	–	16.26	5.32	1–21
Gender					
Female	20	15.5	–	–	–
Male	109	84.5	–	–	–
Age	–	–	62.24	11.25	24–83
Marital status					
Single	8	6.4	–	–	–
Married/in a relationship	92	73.0	–	–	–
Divorced/widowed	26	20.6	–	–	–
Nationality					
Swiss	118	91.5	–	–	–
Other	10	7.8	–	–	–
Children					
0	18	14.3	–	–	–
1	17	13.5	–	–	–
2	56	44.4	–	–	–
3+	35	27.8	–	–	–
Smoking status					
Never smoked	59	48.4	–	–	–
Smoked, quit > 1 year ago	36	29.5	–	–	–
Smoked, quit < 12 months ago	6	4.9	–	–	–
Smoked, quit < 3 months ago	14	11.5	–	–	–
Smokes occasionally	3	2.5	–	–	–
Smokes daily	4	3.3	–	–	–
Most frequent diagnoses					
Ischaemic heart disease	87	67.4	–	–	–
Valve disorder	43	33.3	–	–	–
Aortic aneurysm	14	10.9	–	–	–
Heart failure	23	17.8	–	–	–
Hypertension	45	34.9	–	–	–
Type II diabetes	12	9.3	–	–	–
Number of diagnoses (max. 9)	–	–	6.45	1.71	1–9
Most frequent medications					
Anticoagulants	118	91.5	–	–	–
Statins	99	76.7	–	–	–
Antihypertensives	98	76.0	–	–	–
Antiarrhythmics	22	17.1	–	–	–
Diuretics	53	41.1	–	–	–
Combined preparations	31	24.0	–	–	–
Number of medications (max. 10)	–	–	6.66	2.15	2–10
Body mass index (BMI)	–	–	26.07	4.35	17.43–52.69
MVPA before rehab/hospital stay in minutes/week (self-report)	–	–	294.65	150.70	0–540
Quality of life (MacNew Heart)					
Emotional quality of life	–	–	5.29	1.12	2.55–7.00
Physical quality of life	–	–	5.08	1.11	2.00–7.00
Social quality of life	–	–	5.75	1.13	1.33–7.00

*Note*. *N* = 129 cardiac disease patients. Self-reported moderate-to-vigorous physical activity (MVPA) before rehabilitation or hospital stay was measured the day before the diary phase started with the International Physical Activity Questionnaire (Booth, 2000). Quality of life was measured at the beginning of rehabilitation with the MacNew Heart Disease Quality of Life Questionnaire (MacNew Heart; Höfer, Lim, Guyatt, & Oldridge, 2004). The subscales follow the factorial structure proposed by Bermudez et al. (2021a).

### Measurements

PA and SB were measured objectively using a triaxial accelerometer (ActiGraph, GT3X Monitors, Pensacola, FL, USA). Detailed information about the scoring of the ActiGraph data is described in https://osf.io/4eqdj. The selected outcomes are MVPA, LPA and SB in minutes/day.

All self-report variables were measured using items rated on a six-point Likert scale from *not at all today* – 0 to *extremely today* – 5. *Adjustment disorder symptoms* were assessed using four items adapted for daily measurement and cardiac disease from the Adjustment Disorder New Module 20 (ADNM-20; Lorenz, Bachem, & Maercker, 2016), a screening instrument developed and validated parallel to the ICD-11 diagnostic definition. Two items focused on *preoccupations*: ‘I had to think about my heart disease and its consequences repeatedly today’ and ‘I have to think about my heart disease a lot and this has been a great burden to me today’. Two items addressed *failure to adapt*: ‘I was reluctant today to go to work or to carry out the necessary tasks in everyday life, because of my heart disease and its consequences’ and ‘The heart disease and its consequences affected me strongly today in my personal relationships, my leisure activities or in other important areas of life’. The daily adjustment disorder score was calculated as the mean of the four items. Higher scores represent more severe daily adjustment disorder symptoms. Scores showed an interindividual reliability of *R*_KF_ = 0.996, and an intraindividual reliability of *R*_C_ = 0.68 (Cranford et al., 2006).

Depression and anxiety symptoms were measured using the Patient Health Questionnaire (Löwe et al., 2010) adapted for daily measurement. Following the question ‘How much have you been bothered by the following problems today?’, depression symptoms were assessed with two items: ‘feeling down, depressed or hopeless’ and ‘little interest or pleasure in doing things’ (interindividual correlation *r*_b_ = 0.84, *p* < 0.001; intraindividual correlation *r*_w_ = 0.44, *p* < 0.001). The two items for anxiety were ‘not being able to stop or control worrying’ and ‘feeling nervous, anxious or on edge’ (interindividual correlation *r*_b_ = 0.87, *p* < 0.001; intraindividual correlation *r*_w_ = 0.43, *p* < 0.001). The depression and anxiety symptoms scores were the mean of the two corresponding items with higher scores representing more severe symptoms.

### Statistical analyses

Daily adjustment disorder, depression and anxiety symptoms scores were grand-mean-centred to analyse interindividual associations, and person-mean-centred to investigate the intraindividual level (Bolger & Laurenceau, 2013). These intraindividual predictors were lagged one day to test the lagged hypotheses.

Gaussian multilevel modelling was applied to investigate LPA and SB. Log-transformed negative binomial multilevel modelling was used to investigate MVPA, as it deviated from a normal distribution (Allison, 2012). Notice that this differed from our preregistered zero-inflated Poisson model to follow recommendations for cases with skewed data (Allison, 2012). Incidence rate ratios (*IRR*), calculated as the exponential of the negative binomial regression parameters ( = *e^b^*; Chin & Quddus, 2003), are reported. *IRR*s > 1 indicate a positive association, *IRR*s < 1 imply a negative association (Chin & Quddus, 2003). The difference between *IRR* and 1 can be interpreted as the change in per cent that one unit of the predictor is estimated to have on the outcome (Chin & Quddus, 2003).

The multilevel models were calculated using Bayesian estimation with the R brms package (Buerkner, 2018), which allowed us to include a maximal random effect structure (Barr, Levy, Scheepers, & Tily, 2013). The models included interindividual, same-day intraindividual and lagged intraindividual adjustment disorder, depression and anxiety symptoms. All models were adjusted for the following essential control variables: centred accelerometer *wear time* in minutes, linear *time* in days centred at discharge and centred previous day outcome. The mixed models make use of all available data. With this approach we assume that missing data are missing at random (Bolger & Laurenceau, 2013). Note that no participant was excluded from the main analyses. Other possible control variables were added to the models in sensitivity analyses and are reported in the online Supplementary Table S1. We report the lagged models here as they test both same-day and lagged hypotheses (see online Supplementary Table S2 for same-day only models).

Finally, we included an interaction between the *discharge* variable ( = 0 on days during rehabilitation, = 1 on days after discharge) and each intraindividual predictor to explore the role of context. To keep the models as simple as possible, non-significant interactions were removed. The online Supplementary material reports the models with all interactions in online Supplementary Tables S3–S5.

## Results

The descriptive statistics of all variables, as well as their inter- and intraindividual correlations, are presented in Table 2. The three Bayesian multilevel models (for MVPA, LPA and SB) testing our hypotheses are shown in Table 3.

**Table 2.** Means, standard deviations, inter- and intraindividual correlations of all predictors and outcomes

	Range	*M_b_*	s.d.*_b_*	s.d.*_w_*	*ICC*	1	2	3	4	5	6
1. Adjustment disorder symptoms	[0–5]	1.09	0.99	0.51	0.74	–	0.46***	0.43***	–0.10***	−0.06**	0.05*
2. Depression symptoms	[0–5]	0.60	0.82	0.46	0.65	0.83***	–	0.57***	−0.06**	−0.07***	0.04*
3. Anxiety symptoms	[0–5]	0.57	0.84	0.42	0.69	0.79***	0.89***	–	−0.05**	−0.04*	0.02
4. MVPA	[0–409]	49.26	32.48	28.21	0.49	−0.23**	−0.27**	−0.18*	–	−0.13***	−0.24***
5. LPA	[77–645]	277.06	68.50	63.93	0.49	0.03	−0.04	0.10	0.24**	–	−0.32***
6. Sedentary behaviour	[123–1‘252]	509.18	114.30	87.47	0.56	−0.15	−0.07	−0.16	−0.36***	−0.50***	–

*Note.* Interindividual correlations below diagonal. Intraindividual correlations above diagonal. *M_b_*, interindividual mean; s.d.*_b_*, interindividual standard deviation; s.d.*_w_*, pooled intraindividual standard deviation; *ICC*, intraclass correlation (percentage of variance that is related to interindividual differences); MVPA, moderate to vigorous physical activity in minutes; LPA, light physical activity in minutes.

Significance levels: *p < 0.05; **p < 0.01; ***p < 0.001.

**Table 3.** Bayesian lagged multilevel models

	Outcome MVPA^a^					Outcome LPA^b^				Outcome SB^b^			
			95% CI		*IRR*	*b*	s.e.	95% CI		*b*	s.e.	95% CI	
	*b*	s.e.	LL	UL				LL	UL			LL	UL
Intercept	3.72*	0.08	3.56	3.87	41.27	270.53*	4.42	261.52	279.18	521.95*	5.60	511.00	533.41
Interindividual level													
Adjustment disorder symptoms	−0.09	0.12	−0.33	0.14	0.91	8.49	7.40	−5.63	23.18	−2.00	9.70	−20.94	16.84
Depression symptoms	−0.45*	0.21	−0.88	−0.06	0.64	−39.07*	12.82	−64.52	−14.31	60.57*	16.18	27.90	91.86
Anxiety symptoms	0.24	0.17	−0.08	0.60	1.27	29.98*	11.84	7.56	54.33	−47.84*	15.17	−77.42	−17.49
Same-day intraindividual level													
Adjustment disorder symptoms	−0.23*	0.06	−0.35	−0.11	0.79	−1.47	2.76	−7.01	3.93	7.03*	3.27	0.58	13.70
Adjustment disorder symptoms × discharge (at home)	0.15*	0.07	0.002	0.29	1.16	–	–	–	–	–	–	–	–
Depression symptoms	−0.14*	0.05	−0.24	−0.04	0.87	−9.32*	2.95	−15.33	−3.75	9.79*	3.24	3.55	16.24
Anxiety symptoms	0.06	0.04	−0.03	0.14	1.06	4.07	3.02	−1.85	9.97	−4.18	3.15	−10.44	1.75
Lagged intraindividual level													
Adjustment disorder symptoms	−0.003	0.04	−0.07	0.07	1.00	2.77	2.61	−2.42	7.99	−1.91	2.77	−7.41	3.55
Depression symptoms	−0.04	0.04	−0.12	0.04	0.96	−3.57	2.94	−9.38	2.24	4.05	3.08	−1.99	9.88
Anxiety symptoms	0.05	0.04	−0.03	0.12	1.05	0.005	2.99	−5.67	5.92	−0.94	3.19	−7.2	5.40
Control variables													
Time	0.002	0.004	−0.01	0.01	1.00	1.49*	0.23	1.05	1.95	−1.53*	0.26	−2.05	−1.01
Wear time	0.0002	0.0002	−0.0002	0.0006	1.00	0.28*	0.02	0.24	0.32	0.71*	0.02	0.67	0.74
Previous day outcome	0.005*	0.001	0.003	0.01	1.00	0.17*	0.03	0.12	0.21	0.08*	0.02	0.04	0.11
Discharge (at home)	−0.12*	0.05	−0.23	−0.02	0.88	–	–	–	–	–	–	–	–
Random effects													
Intercept	0.75	0.07	0.62	0.89	–	43.2	3.67	36.55	50.85	60.62	4.61	52.15	70.33
Same-day adjustment disorder	0.07	0.05	0.003	0.18	–	7.92	4.39	0.53	16.6	14.85	4.72	4.76	23.69
Same-day depression symptoms	0.23	0.07	0.09	0.37	–	5.00	3.45	0.23	12.57	6.69	4.35	0.30	16.09
Same-day anxiety symptoms	0.09	0.05	0.005	0.20	–	4.32	3.24	0.19	12.08	4.08	3.14	0.16	11.69
Lagged adjustment disorder	0.10	0.05	0.01	0.20	–	3.44	2.65	0.15	9.78	3.95	2.98	0.17	11.13
Lagged depression symptoms	0.10	0.06	0.005	0.23	–	6.14	3.83	0.32	14.24	4.59	3.35	0.16	12.43
Lagged anxiety symptoms	0.07	0.05	0.003	0.18	–	3.59	2.67	0.14	9.93	3.26	2.51	0.13	9.33
Time	0.02	0.004	0.01	0.03	–	1.61	0.25	1.14	2.12	2.11	0.27	1.59	2.66
Wear time	0.001	0.0004	0.0001	0.002	–	0.11	0.02	0.08	0.15	0.12	0.02	0.08	0.16
Previous day outcome	0.005	0.001	0.003	0.01	–	0.13	0.03	0.08	0.18	0.08	0.03	0.01	0.14

*Note*. *N*, 129 patients; *n*, 2458 days; MVPA, moderate-vigorous physical activity; LPA, light physical activity; SB, sedentary behaviour; *b*, unstandardized regression coefficient; s.e., standard error; *IRR*, incidence rate ratio (i.e. *e^b^*); CI, credible interval; LL, lower limit; UL, upper limit.

Estimates marked with an ‘*’ represent significant results inferred from the 95% CI excluding zero. Note that significance for the random effects cannot be derived from the CI, given that the regression coefficient is the estimated standard deviation (always positive).

a Negative binomial multilevel model.

b Gaussian multilevel model.

### Results for MVPA

Interindividual results showed that patients with higher symptoms of depression engaged in less MVPA throughout the study (H2.1.). Adjustment disorder and anxiety symptoms showed no significant interindividual association with MVPA (H1.1., H3a.1. and H3b.1. not supported). Same-day intraindividual associations showed that on days with higher adjustment disorder (H1.2.) and higher depression (H2.2.) symptoms than usual, patients accumulated less MVPA. As for context, only the interaction between same-day adjustment disorder symptoms and discharge was significant. During rehabilitation, on days with a unit higher adjustment disorder symptoms than usual, MVPA was 21% shorter (*IRR* = 0.79). Back at home, this association was still negative, but significantly less pronounced (MVPA was 8% shorter, *IRR* = 0.92). A model for MVPA including all other (non-significant) interactions with discharge is in online Supplementary Table S3. Finally, lagged associations were all non-significant (hypotheses H1.3., H2.3., H3a.3. and H3b.3. not supported).

### Results for LPA

Patients with lower symptoms of depression (H2.1.) and higher symptoms of anxiety (H3b.1. and refuting H3a.1.) engaged in more LPA. Patients with a unit higher symptoms of depression on average throughout the study engaged in 39.07 min less LPA per day (*b* in Table 3). On days with higher depression symptoms than usual, LPA was 9.32 min shorter (H2.2). No other intraindividual association with LPA was significant (H1.2., H3a.2./H3b.2., H1.3., H2.3., H3a.3./H3b.3. not supported). Also, no significant interactions with discharge emerged (see online Supplementary Table S4).

### Results for SB

No specific hypotheses were formulated for SB. Results showed that patients with higher depression symptoms and lower anxiety symptoms spent more SB time throughout the study. There was no significant interindividual association between adjustment disorder symptoms and SB. At the same-day intraindividual level, adjustment disorder and depression symptoms were positively associated with SB, suggesting that on days with one unit higher symptoms of adjustment disorder than usual, SB was 7.03 min higher. There was no significant association between same-day anxiety symptoms or any of the lagged symptoms and SB. All interactions with discharge proved non-significant (see online Supplementary Table S5).

### Sensitivity analyses

In the sensitivity analyses reported in the online Supplementary material, we controlled for a series of sociodemographic variables (see Supplementary section S1). Patterns of results remained mostly unchanged with the exception of anxiety symptoms, which became significant at the interindividual level for MVPA; and the same-day intraindividual interaction between adjustment disorder symptoms and discharge was rendered non-significant.

Additionally, owing to the high interindividual correlations between the different types of symptoms (see Table 2), we tested models with each type of symptoms individually without adjusting for the others (see online Supplementary Tables S6–S8). Results indicate that anxiety was no longer significantly related to LPA or SB at the interindividual level (online Supplementary Table S6). Adjustment disorder showed a significant negative association with MVPA at the interindividual level (online Supplementary Table S7). All other results remained unchanged.

## Discussion

The study's main goal was to investigate the unique associations of adjustment disorder, depression and anxiety symptoms with the PA and SB of patients during and after inpatient cardiac rehabilitation. Ambulatory assessments were used to analyse associations at the interindividual, same-day intraindividual and lagged intraindividual levels, as well as any differences in these relationships depending on the context in the clinic and after discharge.

Interindividually, we found no significant association of adjustment disorder symptoms with MVPA, LPA or SB. But at the intraindividual level, on days with higher symptoms of adjustment disorder than usual, MVPA was shorter and SB was longer. Perhaps the ICD-11 core symptom of preoccupations offers an explanation as patients might be too immersed in worries to engage in many activities. However, given the concurrent nature of the same-day analyses, the direction of this association is unclear. It is possible that engaging in more MVPA or in less SB throughout the day might lead to a reduction in adjustment disorder symptoms. Supporting this notion are findings regarding the effectiveness of MVPA as an intervention for stress-response disorders (Hegberg, Hayes, & Hayes, 2019). However, there is also evidence for a bidirectional association of stress-related or affective symptoms and PA (Azevedo Da Silva et al., 2012). The concurrent negative association between adjustment disorder symptoms and MVPA was stronger during rehabilitation. Potentially, this might represent a negative influence on cardiac rehabilitation outcomes and cardiovascular prognosis. Future research should further address this. In general, the study's results pertaining adjustment disorder symptoms highlight the importance of such symptoms in cardiac patients over and above depression and anxiety symptoms, and suggest that screening for and treating adjustment disorder during rehabilitation might prove fruitful regarding the PA of patients. Future research should address the prospective long-term association between adjustment disorder symptoms and PA.

Mirroring the consistency in the literature, patients with higher daily symptoms of depression throughout the study engaged in less MVPA and less LPA compared to patients with lower symptoms. Thus, results replicated previous empirical findings (Schuch et al., 2017). A novel contributing factor was the consideration of the intraindividual level in same-day and lagged analyses. Results showed that on days with higher depression symptoms than usual, MVPA and LPA were shorter. Once again the opposite direction or bidirectionality are possible (Azevedo Da Silva et al., 2012). Moreover, no lagged effects were found. These results help to clarify that the negative association between depression symptoms and PA happens on the same day, which contributes to the neglected and complex issue of time dynamics (Scholz, 2019).

There was a substantial interindividual association between depression and SB: according to our data, a patient with the highest possible score of depression symptoms would be expected to engage in more than 6 h more SB per day than a patient with the lowest possible score. In addition, on days with higher symptoms of depression than usual, SB was longer. Given that SB seems to increase cardiovascular risk independent of PA (Wilmot et al., 2012), these results underline the importance of depression symptoms for cardiovascular prognosis.

Depression has also been repeatedly linked to more negative outcomes and cardiac prognosis (Cohen, Edmondson, & Kronish, 2015). Given the importance of regular PA and adequate levels of SB for the treatment of cardiac disease (Halloway et al., 2016; Wilmot et al., 2012), our findings suggest that one possible underlying mechanism of the link between depression and cardiac prognosis might be the amount of PA and SB that patients engage in.

Lastly, in the case of *anxiety*, most studies in the literature found a negative association of anxiety and PA (Azevedo Da Silva et al., 2012; Strine et al., 2005). In our analyses with all symptoms as predictors included, however, patients with higher anxiety symptoms than others engaged in *more* LPA and *less* SB than patients with lower symptoms. It has to be noted, however, that in the sensitivity analyses with anxiety symptoms only as predictors, effects turned non-significant (see online Supplementary material, section 4). Thus, one potential explanation for the direction of the associations might be the high correlations at the interindividual level between anxiety, adjustment disorder and depressive symptoms (see Table 2). Yet, given that previous studies reported positive effects of anxiety symptoms on improvements in exercise capacity and in physical quality of life during rehabilitation (Bermudez et al., 2021a), a second explanation might still be that the strong overlap between depression symptoms, adjustment disorder and anxiety might mask the unique effects that only show when the other constructs are controlled for. Yet, all results should be interpreted very carefully at the interindividual level given the very high interindividual correlations between the different symptoms, and the association between anxiety and PA/SB should surely be investigated further.

The present study adds to the extant literature in different ways and has several strengths. First, taking ICD-11 adjustment disorder (Maercker & Lorenz, 2018) into account is a novel aspect. Second, the intensive-longitudinal design with ambulatory assessments allowed intraindividual analyses and real-time measurements, minimized retrospective bias and increased ecological validity (Reichert et al., 2020). Third, the same-day and lagged analyses contribute to knowledge regarding time dynamics which is sorely lacking (Scholz, 2019). Fourth, MVPA, LPA and SB were all investigated and measured objectively. This is important, because evidence suggests that each type of (in-)activity represents an independent health risk/protection (Amagasa et al., 2018; Wilmot et al., 2012). Finally, possible differences between the time during rehabilitation and after discharge were explored.

The study has some limitations that need to be mentioned. First, our measurements might have caused reactivity (e.g. Baumann et al., 2018). Second, our sample could be prone to selectivity bias. For example, patients with severe affective or stress-response symptoms could have been more likely to decline participation. Indeed, although the complete range of symptoms was included in the study, there were only a few patients with very high symptoms. Associations may have been more pronounced in a sample of patients with severe symptoms. However, intraindividual results point to associations independent of the general level of symptoms of the individual. Thus, patients with general low levels of symptoms could also benefit from interventions due to the fluctuations in symptoms in everyday life. Third, daily adjustment disorder, depression and anxiety symptoms were measured using very few items. Nevertheless, this allowed us to measure them daily, which is an important strength. Finally, given the study design and correlative data it is impossible to infer causality for any of the associations found.

Overall, the results of this study highlight the relevance of adjustment disorder, depression and anxiety symptoms for PA and SB in cardiac patients. The necessity to screen for and treat ICD-11 adjustment disorder and depression symptoms during cardiac rehabilitation is emphasized (O'Donnell, Agathos, Metcalf, Gibson, & Lau, 2019), as this might optimize rehabilitation outcomes and adherence of patients to PA recommendations. Ideally, cardiac rehabilitation programmes could consistently include screening questionnaires, such as ADNM-20 (Lorenz et al., 2016), to identify patients with more severe symptoms. A recent review points to cognitive behavioural therapy, PA itself or pharmacotherapy as viable effective treatments for depression (Jha, Qamar, Vaduganathan, Charney, & Murrough, 2019), which perhaps may also help improve adjustment disorder symptoms.

## Supplementary material

For supplementary material accompanying this paper visit https://doi.org/10.1017/S0033291722003154. click here to view supplementary material
