# Supplementary material for: The role of daily adjustment disorder, depression and anxiety symptoms for the physical activity of cardiac patients
Source: Psychol Med. 2022 Oct 24;53(13):5992–6001. doi: 10.1017/S0033291722003154 (PMC10520595; doi:10.1017/S0033291722003154)
Supplement: Supplementary file 1 [file S0033291722003154sup001.pdf]

Supplementary Material

Daily symptoms of adjustment disorder, depression and anxiety on the physical activity of cardiac patients

Tania Bermudez, Andreas Maercker, Walter Bierbauer, Artur Bernardo, Ruth Fleisch-Silvestri,

Matthias Hermann, Jean-Paul Schmid, & Urte Scholz

Table of contents:

1. Inclusion of all control variables for sensitivity analyses → p. 2
2. Preregistered same-day multilevel models → p. 4
3. Preregistered exploratory analyses → p. 6
4. Multilevel models with anxiety symptoms only → p. 10
5. Multilevel models with adjustment disorder symptoms only → p. 12
6. Multilevel models with depression symptoms only → p. 13
7. Missing values in each measurement point → p. 14

1. Inclusion of all control variables for sensitivity analyses

This section of the supplementary material shows different models for sensitivity analysis, for transparency reasons, or for completeness regarding the registration of the hypotheses and statistical plan prior to the analyses of the data (see [link blinded for review] preregistration).

The following control variables were included for sensitivity analyses:

1) Age: centred at the mean.
2) Gender: coded as 0 for female and 1 for male.
3) Weekday: coded as 1 for weekday and 0 for weekend day = 0.
4) Discharge: coded as 0 for days at the clinic and 1 for days at home after discharge.
5) Low variance: differentiating people with variance in their questionnaire answers across time (=0) from people without variance = 1.
6) Smoking: coded as 0 for non-smokers and as 1 for smokers.
7) Physical quality of life: Score for self-reported physical quality of life at the beginning of cardiac rehabilitation measured with the MacNew Heart Disease Quality of Life Questionnaire (Höfer et al., 2004). Items to form the subscale for physical quality of life were chosen following the proposed factorial structure by Bermudez et al. (2021) and were rated on a 7-point-Likert-scale from 1 (e.g., never) to (e.g., all the time). The score was built as the mean of the items and, thus, could range from 1 to 7. Higher values indicate a higher quality of life.
8) Number of diagnoses: This control variables indicates the number of ICD-10 diagnoses in record at the rehabilitation center (maximum of 9) as a proxy for comorbidity.
9) BMI: Body-Mass-Index.

Table S1 shows the Bayesian lagged multilevel models for each outcome (MVPA, LPA, and SB) and with all control variables for sensitivity analyses.

References

Bermudez, T., Bierbauer, W., Scholz, U., & Hermann, M. (2021). Depression and anxiety in cardiac rehabilitation: Differential associations with changes in exercise capacity and quality of life. *Anxiety, Stress, and Coping*, *35*(2), 204–218. https://doi.org/10.1080/10615806.2021.1952191

Höfer, S., Lim, L., Guyatt, G., & Oldridge, N. (2004). The MacNew Heart disease health-related quality of life instrument: A summary. *Health and Quality of Life Outcomes*, *2*(3), 1–8. https://doi.org/10.1186/1477-7525-2-3

Table S1

Bayesian lagged multilevel models with all control variables for sensitivity analyses

	Outcome MVPA[1]					Outcome LPA[2]				Outcome SB[2]			
	b	*SE*	95% CI		*IRR*	*b*	*SE*	95% CI		*b*	*SE*	95% CI	
			LL	UL				LL	UL			LL	UL
Intercept	4.04*	0.79	2.44	5.55	56.86	373.55*	43.93	288.94	459.04	368.99*	56.94	255.23	478.79
Interindividual level													
Adjustment disorder symptoms	-0.14	0.15	-0.44	0.15	0.87	-2.51	8.33	-19.18	13.81	11.72	11.25	-10.25	33.54
Depression symptoms	-0.74*	0.25	-1.23	-0.27	0.48	-31.25*	13.01	-56.43	-5.95	55.60*	17.28	21.69	89.28
Anxiety symptoms	0.43*	0.20	0.05	0.83	1.53	31.75*	11.98	7.68	54.58	-50.62*	15.49	-81.07	-21.08
Same-day intraindividual level													
Adjustment disorder symptoms	-0.21*	0.06	-0.34	-0.08	0.81	-2.00	2.92	-7.74	3.74	8.23*	3.37	1.68	14.88
Adjustment disorder symptoms* Discharge (at home)	0.10	0.07	-0.05	0.24	1.11	-	-	-	-	-	-	-	-
Depression symptoms	-0.13*	0.05	-0.24	-0.03	0.88	-8.69*	3.04	-14.63	-2.71	9.40*	3.38	2.87	16.08
Anxiety symptoms	0.05	0.04	-0.04	0.13	1.05	3.84	2.99	-2.13	9.73	-4.14	3.22	-10.40	2.20
Lagged intraindividual level													
Adjustment disorder symptoms	-0.02	0.04	-0.10	0.06	0.98	2.99	2.65	-2.29	8.27	-1.76	2.89	-7.36	3.97
Depression symptoms	-0.05	0.04	-0.14	0.04	0.95	-4.13	3.18	-10.32	2.11	3.95	3.13	-2.21	10.15
Anxiety symptoms	0.04	0.04	-0.04	0.13	1.05	0.31	3.12	-5.63	6.55	-0.57	3.08	-6.73	5.38
Control variables													
Time	0.001	0.004	-0.01	0.01	1.00	1.19*	0.30	0.60	1.78	-1.21*	0.32	-1.83	-0.59
Wear time	0.0001	0.0002	-0.0003	0.0005	1.00	0.28*	0.02	0.25	0.31	0.71*	0.02	0.67	0.75
Previous day outcome	0.003*	0.001	0.001	0.005	1.00	0.15*	0.03	0.10	0.20	0.07*	0.02	0.03	0.11
Age	-0.01	0.01	-0.03	0.002	0.99	-0.12	0.40	-0.93	0.66	0.58	0.54	-0.46	1.65
Gender (male)	0.61*	0.24	0.15	1.09	1.84	-29.6*	12.54	-53.36	-4.80	13.98	16.4	-18.78	46.38
Weekday	0.12*	0.04	0.05	0.19	1.13	14.74*	2.73	9.38	20.09	-18.99*	2.86	-24.50	-13.25
Discharge (at home)	-0.09	0.06	-0.20	0.02	0.91	9.37*	4.55	0.78	18.23	-10.46*	4.70	-19.66	-1.37
Low variance	-0.20	0.31	-0.81	0.39	0.82	-5.16	17.48	-40.24	28.65	15.09	24.13	-32.77	62.36
Smoking	-0.16	0.37	-0.88	0.55	0.85	-8.91	19.04	-46.28	28.13	18.50	26.71	-34.56	71.70
Physical quality of life	-0.02	0.07	-0.16	0.13	0.98	-2.09	4.11	-9.96	6.03	4.50	5.63	-6.54	15.79
Number of diagnoses	0.01	0.04	-0.08	0.09	1.01	2.34	2.43	-2.41	7.24	-1.82	3.23	-8.07	4.63
BMI	-0.03	0.02	-0.08	0.01	0.97	-3.65*	1.18	-5.93	-1.34	5.47*	1.55	2.37	8.49
Random effects													
Intercept	0.80	0.08	0.66	0.96	-	39.18	3.77	32.39	47.14	56.12	4.70	47.53	65.94
Same-day adjustment disorder	0.08	0.05	0.004	0.19	-	7.11	4.4	0.37	16.39	14.05	4.96	3.37	23.42
Same-day depression symptoms	0.25	0.07	0.09	0.39	-	5.49	3.65	0.27	13.6	8.29	4.52	0.58	17.39
Same-day anxiety symptoms	0.08	0.05	0.004	0.20	-	3.8	2.88	0.15	10.76	4.00	3.08	0.10	11.27
Lagged adjustment disorder symptoms	0.11	0.06	0.01	0.23	-	3.46	2.61	0.13	9.77	3.9	2.90	0.17	10.67
Lagged depression symptoms	0.14	0.07	0.01	0.27	-	7.01	4.25	0.39	15.96	5.37	3.81	0.25	14.2
Lagged anxiety symptoms	0.09	0.06	0.01	0.21	-	3.28	2.47	0.11	9.32	3.21	2.47	0.12	9.14
Time	0.02	0.004	0.02	0.03	-	1.59	0.26	1.08	2.12	1.95	0.26	1.46	2.49
Wear time	0.001	0.0003	0.00003	0.001	-	0.08	0.03	0.02	0.14	0.09	0.03	0.04	0.14
Previous day outcome	0.003	0.001	0.001	0.01	-	0.14	0.03	0.08	0.19	56.12	4.70	47.53	65.94

Note. *N* = 114 patients. *n* = 2'170 days. MVPA = moderate-vigorous physical activity. LPA = light physical activity. SB = sedentary behaviour. *b* = unstandardized regression coefficient. *SE* = standard error. *IRR* = incidence rate ratio (i.e., e^b). CI = credible interval. LL = lower limit. UL = upper limit. [1] Negative binomial multilevel model. [2] Gaussian multilevel model. Estimates marked with an "*" represent significant results inferred from the 95% CI excluding zero. Note that significance for the random effects cannot be derived from the CI, given that the regression coefficient is the estimated standard deviation (always positive).

2. Preregistered same-day multilevel models

The registered statistical analyses also included same-day only models. These models are reported in Table S2 and include interindividual and same-day intraindividual predictors (adjustment disorder, depression, and anxiety symptoms), as well as essential control variables (wear time and time).

Table S2

Bayesian same-day multilevel models

	Outcome MVPA[1]					Outcome LPA[2]				Outcome SB[2]			
	b	*SE*	95% CI		*IRR*	*b*	*SE*	95% CI		*b*	*SE*	95% CI	
			LL	UL				LL	UL			LL	UL
Intercept	3.57*	0.09	3.40	3.73	35.41	267.68*	5.01	257.77	277.38	522.24*	6.14	510.22	534.08
Interindividual level													
Adjustment disorder symptoms	-0.16	0.16	-0.47	0.15	0.85	7.46	8.23	-8.7	23.9	1.47	10.22	-18.79	21.42
Depression symptoms	-0.84*	0.25	-1.34	-0.34	0.43	-40.13*	14.24	-67.95	-12.33	54.28*	17.54	20.84	89.45
Anxiety symptoms	0.52*	0.23	0.08	0.97	1.69	33.85*	12.9	8.66	59.19	-48.00*	15.67	-78.92	-17.68
Same-day intraindividual level													
Adjustment disorder symptoms	-0.13*	0.03	-0.20	-0.06	0.88	-0.78	2.66	-5.97	4.47	6.28*	3.13	0.09	12.4
Depression symptoms	-0.11*	0.05	-0.21	-0.03	0.89	-7.57*	2.72	-12.92	-2.28	8.43*	3.06	2.48	14.49
Anxiety symptoms	0.04	0.04	-0.04	0.11	1.04	2.55	2.73	-2.81	7.9	-2.61	2.89	-8.32	3.09
Control variables													
Time	-0.01	0.003	-0.01	0.0003	0.99	1.84*	0.25	1.36	2.33	-1.52*	0.27	-2.06	-0.98
Wear time	0.0003	0.0002	-0.0001	0.001	1.00	0.28*	0.02	0.25	0.31	0.71*	0.02	0.68	0.75
Random effects													
Intercept	0.93	0.07	0.81	1.08	0.93	53.45	3.83	46.52	61.55	66.88	4.61	58.54	76.49
Same-day adjustment disorder symptoms	0.08	0.05	0.004	0.19	0.08	9.79	4.21	1.11	17.55	15.88	4.08	7.45	23.69
Same-day depression symptoms	0.23	0.06	0.11	0.35	0.23	5.50	3.67	0.27	13.64	8.91	4.54	0.80	18.01
Same-day anxiety symptoms	0.07	0.05	0.004	0.18	0.07	3.70	2.82	0.14	10.45	3.77	2.82	0.13	10.48
Time	0.03	0.003	0.02	0.04	0.03	2.23	0.22	1.81	2.69	2.50	0.24	2.05	3.00
Wear time	0.001	0.0004	0.0001	0.001	0.001	0.11	0.02	0.07	0.14	0.11	0.02	0.08	0.15

Note. *N* = 129 patients. *n* = 2’845 days. MVPA = moderate-vigorous physical activity. LPA = light physical activity. SB = sedentary behaviour. *b* = unstandardized regression coefficient. *SE* = standard error. *IRR* = incidence rate ratio (i.e., e^b). CI = credible interval. LL = lower limit. UL = upper limit. [1] Negative binomial multilevel model. [2] Gaussian multilevel model. Estimates marked with an “*” represent significant results inferred from the 95% CI excluding zero. Note that significance for the random effects cannot be derived from the CI, given that the regression coefficient is the estimated standard deviation (always positive).

3. Preregistered exploratory analyses

Table S3 reports the lagged multilevel model with MVPA as outcome and the inclusion of all exploratory analyses, that is, the interaction of the discharge variable with each intraindividual predictor. Tables S4 and S5 show the same exploratory analyses, but for the outcomes light physical activity (LPA) and sedentary behaviour (SB), accordingly.

Table S3

Bayesian lagged multilevel model with MVPA as outcome and all exploratory interactions with after rehab

	Outcome MVPA[1]				
	b	*SE*	95% CI		IRR
			LL	UL	
Intercept	3.74*	0.08	3.58	3.89	42.10
Interindividual level					
Adjustment disorder symptoms	-0.09	0.12	-0.32	0.13	0.91
Depression symptoms	-0.43*	0.20	-0.85	-0.06	0.65
Anxiety symptoms	0.23	0.17	-0.09	0.58	1.26
Same-day intraindividual level					
Adjustment disorder symptoms	-0.27*	0.07	-0.40	-0.14	0.76
Adjustment disorder symptoms * Discharge	0.20*	0.08	0.04	0.35	1.22
Depression symptoms	-0.04	0.09	-0.22	0.14	0.96
Depression symptoms * Discharge	-0.14	0.10	-0.34	0.06	0.87
Anxiety symptoms	0.06	0.08	-0.09	0.21	1.06
Anxiety symptoms * Discharge	-0.01	0.09	-0.18	0.17	0.99
Lagged intraindividual level					
Adjustment disorder symptoms	-0.01	0.06	-0.13	0.12	0.99
Adjustment disorder symptoms * Discharge	0.00	0.08	-0.14	0.15	1.00
Depression symptoms	0.03	0.08	-0.13	0.20	1.03
Depression symptoms * Discharge	-0.09	0.10	-0.28	0.09	0.91
Anxiety symptoms	0.03	0.08	-0.12	0.19	1.03
Anxiety symptoms * Discharge	0.02	0.09	-0.16	0.20	1.02
Control variables					
Time	0.002	0.004	-0.01	0.01	1.00
Wear time	0.0002	0.0002	-0.0002	0.0006	1.00
Previous day outcome	0.005*	0.001	0.003	0.01	1.00
Discharge (at home)	-0.13*	0.05	-0.24	-0.03	0.87
Random effects					
Intercept	0.74	0.07	0.62	0.88	-
Same-day adjustment disorder symptoms	0.07	0.05	0.00	0.18	-
Same-day depression symptoms	0.25	0.07	0.11	0.39	-
Same-day anxiety symptoms	0.09	0.05	0.01	0.20	-
Lagged adjustment disorder symptoms	0.09	0.05	0.01	0.20	-
Lagged depression symptoms	0.09	0.06	0.01	0.22	-
Lagged anxiety symptoms	0.07	0.05	0.00	0.18	-
Time	0.02	0.004	0.01	0.03	-
Wear time	0.001	0.0004	0.0001	0.002	-
Previous day outcomes	0.005	0.001	0.003	0.01	-

Note. *N* = 129 patients. *n* = 2’458 days. MVPA = moderate-vigorous physical activity. *b* = unstandardized regression coefficient. *SE* = standard error. *IRR* = incidence rate ratio (i.e., e^b). CI = credible interval. LL = lower limit. UL = upper limit. [1] Negative binomial multilevel model. [2] Gaussian multilevel model. Estimates marked with an “*” represent significant results inferred from the 95% CI excluding zero. Note that significance for the random effects cannot be derived from the CI, given that the regression coefficient is the estimated standard deviation (always positive).

Table S4

Bayesian lagged multilevel model with LPA as outcome and all exploratory interactions with after rehab

	Outcome LPA			
	b	*SE*	95% CI	
			LL	UL
Intercept	266.07*	4.91	256.26	275.56
Interindividual level				
Adjustment disorder symptoms	8.31	7.64	-6.94	23.44
Depression symptoms	-40.64*	13.12	-66.17	-15.31
Anxiety symptoms	31.58*	12.05	8.34	55.28
Same-day intraindividual level				
Adjustment disorder symptoms	-0.99	5.09	-10.86	8.76
Adjustment disorder symptoms * Discharge	-0.71	5.91	-12.15	10.82
Depression symptoms	-16.01*	6.19	-27.92	-3.81
Depression symptoms * Discharge	8.53	6.98	-5.52	22.52
Anxiety symptoms	9.32	5.68	-1.72	20.20
Anxiety symptoms * Discharge	-6.99	6.67	-20.24	5.89
Lagged intraindividual level				
Adjustment disorder symptoms	-2.25	4.78	-11.48	7.13
Adjustment disorder symptoms * Discharge	6.81	5.74	-4.42	18.12
Depression symptoms	-10.84	6.12	-22.44	0.88
Depression symptoms * Discharge	8.89	6.90	-4.26	22.47
Anxiety symptoms	6.67	5.61	-4.32	17.31
Anxiety symptoms * Discharge	-8.94	6.53	-21.85	3.62
Control variables				
Time	1.22*	0.28	0.68	1.78
Wear time	0.28*	0.02	0.25	0.31
Previous day outcome	0.16*	0.02	0.11	0.20
Discharge (at home)	8.30*	4.40	-0.25	16.83
Random effects				
Time	44.04	3.81	37.17	52.00
Same-day adjustment disorder symptoms	7.99	4.45	0.48	16.71
Same-day depression symptoms	5.44	3.7	0.26	13.67
Same-day anxiety symptoms	4.55	3.32	0.18	12.23
Lagged adjustment disorder symptoms	3.34	2.54	0.13	9.37
Lagged depression symptoms	5.63	3.66	0.28	13.46
Lagged anxiety symptoms	3.54	2.72	0.13	10.27
Time	1.61	0.26	1.11	2.13
Wear time	0.11	0.02	0.08	0.16
Previous day outcomes	0.13	0.03	0.07	0.18

Note. *N* = 129 patients. *n* = 2’458 days. LPA = light physical activity. *b* = unstandardized regression coefficient. *SE* = standard error. CI = credible interval. LL = lower limit. UL = upper limit. [1] Negative binomial multilevel model. [2] Gaussian multilevel model. Estimates marked with an “*” represent significant results inferred from the 95% CI excluding zero. Note that significance for the random effects cannot be derived from the CI, given that the regression coefficient is the estimated standard deviation (always positive).

Table S5

Bayesian lagged multilevel model with SB as outcome and all exploratory interactions with after rehab

	Outcome SB			
	b	*SE*	95% CI	
			LL	UL
Intercept	527.11*	6.19	514.75	539.26
Interindividual level				
Adjustment disorder symptoms	-1.11	9.77	-20.34	17.95
Depression symptoms	61.71*	16.53	29.08	93.45
Anxiety symptoms	-49.23*	15.17	-78.52	-19.68
Same-day intraindividual level				
Adjustment disorder symptoms	8.42	5.59	-2.65	19.49
Adjustment disorder symptoms * Discharge	-1.60	6.40	-14.18	11.00
Depression symptoms	16.90*	6.66	3.90	29.95
Depression symptoms * Discharge	-8.89	7.47	-23.43	5.37
Anxiety symptoms	-9.01	5.86	-20.48	2.74
Anxiety symptoms * Discharge	6.42	6.93	-7.00	19.98
Lagged intraindividual level				
Adjustment disorder symptoms	1.99	5.18	-8.17	12.20
Adjustment disorder symptoms * Discharge	-5.14	6.08	-16.68	6.72
Depression symptoms	12.41	6.47	-0.07	24.85
Depression symptoms * Discharge	-10.40	7.33	-24.61	3.78
Anxiety symptoms	-8.08	6.09	-20.17	3.58
Anxiety symptoms * Discharge	9.50	7.09	-4.24	23.61
Control variables				
Time	-1.21*	0.32	-1.83	-0.58
Wear time	0.71*	0.02	0.67	0.74
Previous day outcome	0.08*	0.02	0.04	0.11
Discharge (at home)	-9.07*	4.47	-17.98	-0.40
Random effects				
Time	61.28	4.78	52.69	71.12
Same-day adjustment disorder symptoms	14.22	4.86	4.09	23.21
Same-day depression symptoms	7.31	4.52	0.38	16.8
Same-day anxiety symptoms	3.89	2.97	0.14	10.94
Lagged adjustment disorder symptoms	4.07	3.01	0.19	11.12
Lagged depression symptoms	4.62	3.42	0.21	12.73
Lagged anxiety symptoms	3.35	2.51	0.17	9.32
Time	2.09	0.26	1.6	2.64
Wear time	0.11	0.02	0.07	0.16
Previous day outcomes	0.08	0.03	0.01	0.14

Note. *N* = 129 patients. *n* = 2’458 days. SB = sedentary behaviour. *b* = unstandardized regression coefficient. *SE* = standard error. CI = credible interval. LL = lower limit. UL = upper limit. [1] Negative binomial multilevel model. [2] Gaussian multilevel model. Estimates marked with an “*” represent significant results inferred from the 95% CI excluding zero. Note that significance for the random effects cannot be derived from the CI, given that the regression coefficient is the estimated standard deviation (always positive).

4. Multilevel models with only anxiety symptoms

Given the surprising direction of the associations of anxiety symptoms found in the models reported in the main article (see Table 3), we decided to run the models including anxiety symptoms only. In Table S6, we report three multilevel models with anxiety symptoms (without adjustment disorder and depression symptoms) and essential control variables only. A model is reported for each type of activity (MVPA, LPA, and SB) as outcome. All associations of anxiety symptoms were rendered non-significant. Multicollinearity is a likely explanation, given the very high icorrelations between the different types of symptoms (see Table 2 in the main article). This would only apply to the interindividual level where correlations between the different types of symptoms were >.70. At the intraindividual level, correlations were not as high to expect multicollinearity. A second explanation might still be that the strong overlap between depression symptoms, adjustment disorder symptoms and anxiety symptoms might mask the unique effects that only show when the other constructs are controlled for.

Table S6

Bayesian lagged multilevel models with only anxiety symptoms

	Outcome MVPA[1]					Outcome LPA[2]				Outcome SB[2]			
	b	*SE*	95% CI		*IRR*	*b*	*SE*	95% CI		*b*	*SE*	95% CI	
			LL	UL				LL	UL			LL	UL
Intercept	3.74*	0.07	3.60	3.88	42.02	270.66*	4.53	261.74	279.56	522.23*	6.06	510.36	534.07
Interindividual level													
Anxiety symptoms	-0.13	0.07	-0.27	0.01	0.88	2.13	5.05	-7.95	12.09	4.07	6.97	-9.77	17.66
Same-day intraindividual level													
Anxiety symptoms	-0.06	0.04	-0.13	0.02	0.95	-1.34	2.60	-6.36	3.85	4.19	2.72	-1.15	9.53
Lagged intraindividual level													
Anxiety symptoms	0.01	0.03	-0.06	0.08	1.01	-1.44	2.42	-6.11	3.30	1.70	2.56	-3.33	6.69
Control variables													
Time	-0.002	0.003	-0.01	0.004	1.00	1.48*	0.23	1.03	1.94	-1.56*	0.27	-2.09	-1.04
Wear time	0.0002	0.0002	-0.0002	0.0006	1.00	0.28*	0.02	0.25	0.32	0.70*	0.02	0.67	0.74
Previous day outcome	0.01*	0.001	0.005	0.01	1.01	0.17*	0.02	0.12	0.22	0.08*	0.02	0.04	0.11
Random effects													
Intercept	0.72	0.07	0.60	0.86	0.72	45.09	3.81	38.16	53.13	64.96	4.83	56.14	75.16
Same-day anxiety symptoms	0.13	0.05	0.02	0.23	0.13	5.96	3.96	0.28	14.54	5.85	3.95	0.25	14.68
Lagged anxiety symptoms	0.08	0.05	0.00	0.19	0.08	3.48	2.66	0.11	9.78	3.16	2.40	0.12	8.91
Time	0.02	0.004	0.01	0.03	0.02	1.60	0.26	1.11	2.12	2.13	0.26	1.63	2.67
Wear time	0.001	0.0004	0.0001	0.002	0.001	0.11	0.02	0.08	0.15	0.11	0.02	0.07	0.16
Previous day outcome	0.01	0.001	0.005	0.01	0.01	0.13	0.03	0.07	0.18	0.07	0.03	0.01	0.13

Note. *N* = 129 patients. *n* = 2’458 days. MVPA = moderate-vigorous physical activity. LPA = light physical activity. SB = sedentary behaviour. *b* = unstandardized regression coefficient. *SE* = standard error. *IRR* = incidence rate ratio (i.e., e^b). CI = credible interval. LL = lower limit. UL = upper limit. [1] Negative binomial multilevel model. [2] Gaussian multilevel model. Estimates marked with an “*” represent significant results inferred from the 95% CI excluding zero. Note that significance for the random effects cannot be derived from the CI, given that the regression coefficient is the estimated standard deviation (always positive).

5. Multilevel models with only adjustment disorder symptoms

Table S7

Bayesian lagged multilevel models with only adjustment disorder symptoms

	Outcome MVPA[1]					Outcome LPA[2]				Outcome SB[2]			
	b	*SE*	95% CI		*IRR*	*b*	*SE*	95% CI		*b*	*SE*	95% CI	
			LL	UL				LL	UL			LL	UL
Intercept	3.76*	0.08	3.61	3.91	43.07	270.80*	4.55	261.93	279.68	521.65*	6.08	509.55	533.53
Interindividual level													
Adjustment disorder symptoms	-0.17*	0.07	-0.31	-0.04	0.84	0.71	4.04	-7.21	8.60	8.73	5.66	-2.30	19.86
Same-day intraindividual level													
Adjustment disorder symptoms	-0.24*	0.06	-0.36	-0.12	0.79	-3.63	2.60	-8.68	1.53	9.48*	3.14	3.19	15.59
Adjustment disorder symptoms * Discharge (at home)	0.13	0.07	-0.01	0.27	1.14	-	-	-	-	-	-	-	-
Lagged intraindividual level													
Adjustment disorder symptoms	-0.002	0.03	-0.07	0.06	1.00	1.20	2.22	-3.14	5.54	-0.43	2.46	-5.25	4.43
Control variables													
Time	0.003	0.004	-0.004	0.01	1.00	1.49*	0.23	1.04	1.94	-1.53*	0.27	-2.07	-1.01
Wear time	0.0002	0.0002	-0.0002	0.001	1.00	0.28*	0.02	0.25	0.32	0.70*	0.02	0.67	0.74
Previous day outcome	0.01*	0.001	0.004	0.01	1.01	0.17*	0.03	0.12	0.22	0.08*	0.02	0.04	0.11
Discharge (at home)	-0.13*	0.05	-0.23	-0.02	0.88	-	-	-	-	-	-	-	-
Random effects													
Intercept	0.74	0.07	0.62	0.88	-	45.70	3.92	38.53	53.85	64.97	4.81	56.22	74.99
Same-day adjustment disorder symptoms	0.12	0.05	0.01	0.22	-	10.46	4.34	1.30	18.54	16.67	4.17	8.38	24.91
Lagged adjustment disorder symptoms	0.12	0.06	0.01	0.23	-	3.13	2.45	0.12	9.13	4.33	3.03	0.19	11.18
Time	0.02	0.004	0.01	0.03	-	1.59	0.26	1.11	2.11	2.11	0.26	1.61	2.65
Wear time	0.001	0.0003	0.0002	0.002	-	0.12	0.02	0.08	0.16	0.12	0.02	0.08	0.16
Previous day outcome	0.01	0.001	0.004	0.01	-	0.13	0.03	0.07	0.18	0.07	0.03	0.01	0.13

Note. *N* = 129 patients. *n* = 2’458 days. MVPA = moderate-vigorous physical activity. LPA = light physical activity. SB = sedentary behaviour. *b* = unstandardized regression coefficient. *SE* = standard error. *IRR* = incidence rate ratio (i.e., e^b). CI = credible interval. LL = lower limit. UL = upper limit. [1] Negative binomial multilevel model. [2] Gaussian multilevel model. Estimates marked with an “*” represent significant results inferred from the 95% CI excluding zero. Note that significance for the random effects cannot be derived from the CI, given that the regression coefficient is the estimated standard deviation (always positive).

6. Multilevel models with only depression symptoms

Table S8

Bayesian lagged multilevel models with only depression symptoms

	Outcome MVPA[1]					Outcome LPA[2]				Outcome SB[2]			
	b	*SE*	95% CI		*IRR*	*b*	*SE*	95% CI		*b*	*SE*	95% CI	
			LL	UL				LL	UL			LL	UL
Intercept	3.71*	0.07	3.57	3.85	40.97	270.40*	4.50	261.56	279.07	522.59*	5.93	510.95	534.10
Interindividual level													
Depression symptoms	-0.25*	0.09	-0.43	-0.08	0.78	-4.26	4.80	-13.65	5.23	16.87*	6.76	3.29	30.09
Same-day intraindividual level													
Depression symptoms	-0.16*	0.05	-0.25	-0.06	0.85	-7.99*	2.48	-12.80	-3.17	11.15*	2.93	5.42	16.98
Lagged intraindividual level													
Depression symptoms	-0.02	0.03	-0.09	0.05	0.98	-1.90	2.47	-6.70	2.90	2.86	2.55	-2.15	7.77
Control variables													
Time	-0.003	0.003	-0.01	0.003	1.00	1.50*	0.23	1.06	1.96	-1.59*	0.26	-2.11	-1.07
Wear time	0.0002	0.0002	-0.0002	0.0006	1.00	0.28*	0.02	0.25	0.31	0.71*	0.02	0.67	0.74
Previous day outcome	0.01	0.001	0.004	0.01	1.01	0.17*	0.02	0.12	0.22	0.08*	0.02	0.04	0.11
Random effects													
Intercept	0.71	0.06	0.59	0.84	0.71	45.24	3.78	38.11	53.05	64.01	4.85	55.24	74.17
Same-day depression symptoms	0.26	0.06	0.14	0.39	0.26	5.89	3.78	0.30	13.90	11.38	4.42	2.22	19.92
Lagged depression symptoms	0.09	0.06	0.005	0.22	0.09	6.78	4.03	0.35	15.14	4.63	3.38	0.19	12.51
Time	0.02	0.004	0.01	0.03	0.02	1.62	0.26	1.14	2.14	2.15	0.27	1.65	2.69
Wear time	0.001	0.0004	0.0001	0.002	0.001	0.11	0.02	0.08	0.15	0.11	0.02	0.07	0.16
Previous day outcome	0.01	0.001	0.004	0.01	0.01	0.13	0.03	0.07	0.18	0.07	0.03	0.01	0.12

Note. *N* = 129 patients. *n* = 2’458 days. MVPA = moderate-vigorous physical activity. LPA = light physical activity. SB = sedentary behaviour. *b* = unstandardized regression coefficient. *SE* = standard error. *IRR* = incidence rate ratio (i.e., e^b). CI = credible interval. LL = lower limit. UL = upper limit. [1] Negative binomial multilevel model. [2] Gaussian multilevel model. Estimates marked with an “*” represent significant results inferred from the 95% CI excluding zero. Note that significance for the random effects cannot be derived from the CI, given that the regression coefficient is the estimated standard deviation (always positive).

7. Missing values in each measurement point

Table S10

Valid and missing values in same-day and lagged models

	Valid in same-day models		Missing in same-day models		Valid in lagged models		Missing in lagged models	
Day	*n*	%	*n*	%	*n*	%	*n*	%
-7	39	30.23%	90	69.77%	0	0.00%	129	100.00%
-6	61	47.29%	68	52.71%	34	26.36%	95	73.64%
-5	89	68.99%	40	31.01%	57	44.19%	72	55.81%
-4	97	75.19%	32	24.81%	86	66.67%	43	33.33%
-3	113	87.60%	16	12.40%	93	72.09%	36	27.91%
-2	115	89.15%	14	10.85%	104	80.62%	25	19.38%
-1	117	90.70%	12	9.30%	105	81.40%	24	18.60%
0	117	90.70%	12	9.30%	109	84.50%	20	15.50%
1	104	80.62%	25	19.38%	96	74.42%	33	25.58%
2	113	87.60%	16	12.40%	96	74.42%	33	25.58%
3	106	82.17%	23	17.83%	99	76.74%	30	23.26%
4	110	85.27%	19	14.73%	98	75.97%	31	24.03%
5	105	81.40%	24	18.60%	96	74.42%	33	25.58%
6	105	81.40%	24	18.60%	94	72.87%	35	27.13%
7	107	82.95%	22	17.05%	96	74.42%	33	25.58%
8	103	79.84%	26	20.16%	94	72.87%	35	27.13%
9	104	80.62%	25	19.38%	95	73.64%	34	26.36%
10	107	82.95%	22	17.05%	95	73.64%	34	26.36%
11	102	79.07%	27	20.93%	96	74.42%	33	25.58%
12	98	75.97%	31	24.03%	85	65.89%	44	34.11%
13	99	76.74%	30	23.26%	83	64.34%	46	35.66%
14	98	75.97%	31	24.03%	88	68.22%	41	31.78%
15	98	75.97%	31	24.03%	84	65.12%	45	34.88%
16	94	72.87%	35	27.13%	83	64.34%	46	35.66%
17	91	70.54%	38	29.46%	81	62.79%	48	37.21%
18	94	72.87%	35	27.13%	79	61.24%	50	38.76%
19	94	72.87%	35	27.13%	82	63.57%	47	36.43%
20	86	66.67%	43	33.33%	79	61.24%	50	38.76%
21	79	61.24%	50	38.76%	71	55.04%	58	44.96%

Note. Negative days represent days at the rehabilitation clinic. Day 0 is the discharge day. Positive days represent days at home after discharge.
